# The impact of a qualified medical documentation assistant on trauma room management

**DOI:** 10.1007/s00068-020-01513-y

**Published:** 2020-10-06

**Authors:** Benjamin Lucas, Sophie-Cecil Mathieu, Gerald Pliske, Wiebke Schirrmeister, Martin Kulla, Felix Walcher

**Affiliations:** 1 Department of Trauma Surgery, Otto-Von-Guericke University Magdeburg, Leipziger Str. 44, 39120 Magdeburg, Germany; 2 Department of Orthopaedic Surgery, Otto-Von-Guericke University Magdeburg, 39120 Magdeburg, Germany; 3 Department of Anaesthesiology, Intensive Care Medicine, Emergency Medicine and Pain Therapy, Bundeswehrhospital Ulm, Oberer Eselsberg 40, 89081 Ulm, Germany

**Keywords:** Documentation, Emergency medicine, Trauma registry, Trauma room

## Abstract

**Purpose:**

To improve quality of trauma room management, intra- and inter-hospital benchmarking are important tools. However, primary data quality is crucial for benchmarking reliability. In this study, we analyzed the effect of a medical documentation assistant on documentation completeness in trauma room management in comparison to documentation by physicians involved in direct patient treatment.

**Methods:**

We included all patients treated in the trauma room from 2016/01/01 to 2016/12/31 that were documented with the trauma module of the German Emergency Department Medical Record V2015.1. We divided the data into documentation by medical documentation assistant (DA, 07:00 to 17:00), physician in daytime (PD, 07:00 to 17:00), and physician at night (PN, 17:00 to 07:00). Data were analyzed for completeness (primary outcome parameter) as well as diagnostic intervals.

**Results:**

There was a significant increase in complete recorded data for DA (74.5%; IQR 14.5%) compared to PD (26.9%; IQR 18.7%; *p* < 0.001) and PN (30.8%; IQR 18.9; *p* < 0.001). The time to whole-body computed tomography (WBCT) significantly decreased for DA (19 min; IQR 8.3) compared to PD (24 min; IQR 12.8; *p* = 0.007) or PN (24.5 min; IQR 10.0; *p* = 0.001).

**Conclusion:**

In presence of a qualified medical documentation assistant, data completeness and time to WBCT improved significantly. Therefore, utilizing a professional DA in the trauma room appears beneficial for data quality and time management.

## Introduction

For severely injured patients, effective diagnostics and treatment in the trauma room are crucial to the course of treatment and outcome of the patient. In recent decades, several improvements have been made to prehospital and early in-hospital treatment. Accordingly, the survival rate of severely injured patients has improved from 63 to 78% [1, 2].

One important factor for improving treatment effectiveness and quality is the use of intra- and inter-hospital benchmarking [1]. The inter-hospital quality assessment of the German Trauma Society (TraumaRegister DGU^®^; TR-DGU) started with six German hospitals in 1993. Since then, the TR-DGU has increased greatly in scope. Currently, more than 600 hospitals from 11 countries are participating [3, 4]. Moreover, for German hospitals, participating in this registry is one of the requirements for the hospital’s certification as a trauma center (TraumaZentrum DGU^®^).

For inter-hospital benchmarking, several quality indicators have been evaluated for inclusion in the TR-DGU [5, 6]. Thus, the quality of the documentation of trauma room diagnostics and treatment is crucial to ensure data quality. As such, standardized and structured documentation is necessary. In this respect, the German Interdisciplinary Association of Intensive and Emergency Care (DIVI) evaluated the German Emergency Department Medical Record (GEDMR). This documentation standard integrates the content of the TR-DGU, which avoids redundancies in the documentation process [7].

Ziprian et al. [8] evaluated data quality of 5409 TR-DGU treatment cases. There were several discrepancies, even for well-evaluated quality indicators such as the time to whole-body computed tomography (WBCT) [5, 8]. That is, initial documentation quality in the trauma room appears to be a major issue. In the current study, the primary outcome parameter was completeness of TR-DGU documentation. The aim of the study was to evaluate the impact of a medical documentation assistant (DA) on documentation completeness in the trauma room in comparison to documentation by physicians involved in direct patient treatment. We expected a better documentation completeness by DA.

## Materials and methods

This single-center study complied with the Helsinki Declaration (October 2013) and was approved by the Ethics Committee of the University of Magdeburg, Germany. The need to obtain written patient consent was waived by the Institutional Review Board, as the study strictly utilized anonymized data obtained from patient treatment and the trauma team. The STrengthening the Reporting of OBservational studies in Epidemiology (STROBE) recommendations were applied with additional focus on study design [9]. Regarding the trauma room treatment, advanced trauma life support (ATLS) is practiced in our institution. The trauma room team was in same constellation in any of the included treatment cases. A medical documentation assistant (DA) was available between 07:00 and 17:00 on weekdays. The DA was a study nurse with a special training in medical documentation. Besides the medical documentation assistant, the trauma room team was the same at this time on weekdays, weekends, and public holidays. Thus, we divided the data in relation to the documentation assistant into three groups a priori: medical documentation assistant (DA, 07:00 to 17:00), physician in daytime (PD, 07:00 to 17:00) and physician at night (PN, 17:00 to 07:00).

### Trauma room documentation and item analysis

We included all trauma room patients of the University Hospital Magdeburg with trauma room documentation in the year 2016. Documentation of these patients used version 2015.1 of the trauma module of the GEDMR [10–12]. In our trauma room setting, documentation is carried out by the physicians of service (trauma surgery) or the DA. As the primary outcome parameter, we analyzed the relative frequency of all items that were necessary for input in the TR-DGU that were not automatically acquired by during patient admission. These items were grouped into patient core data, prehospital data, and trauma room data (Table 1). The TR data of all three groups were compared: items were assessed as “not filled,” “incompletely filled”, or “primary complete”. If specific items did not need to be collected because the diagnostic intervention or treatment was not performed, these items were coded as “not necessary” to prevent bias in relative frequencies. Furthermore, such items were excluded from statistical analysis [13]. As secondary outcome parameters, we analyzed diagnostic and treatment intervals in trauma room, such as the time to whole-body computed tomography (WBCT).

**Table 1:** Data items

Patient core data	Prehospital data	Trauma room data
Accident date	Time of arrival at the scene	**Arterial blood pressure**
Accident time	Type of transportation	Respiratory rate
Cause of accident	**Arterial blood pressure**	**Hemoglobin value**
Accident mechanism	Respiratory rate	**Blood coagulation parameters**
Type of accident	Performance of capnography	**Base excess**
**American Society of Anesthesiologists (ASA) physical status prior to the accident**	**Glasgow coma scale**	Performance of FAST
Anticoagulation	**Pupillary reflex**	Time to FAST
referral from another emergency department	Administered volume	Time to X-ray of the chest
	Airway management	Time to X-ray of the pelvis
	**Cardiopulmonary resuscitation**	Time to cranial CT
	Administration of tranexamic acid	Time to WBCT
		**Time to emergency operations**
		Administration of blood preservation
		**Time of admittance to the emergency department**

For analysis of documentation completeness, we chose the data items of the trauma module of the GEDMR that were mandatory in the TR-DGU. These items were grouped into patient core data, prehospital data, and trauma room data. Items that were specifically used from TR-DGU for documentation completeness are highlighted in bold

### Statistical analysis

SPSS Statistics 25 (IBM, Armonk, USA) was used for statistical analysis. All data are presented as mean ± standard deviation (SD) for normally distributed variables and as medians for variables with non-normal distributions. Normal distributions were verified by Kolmogorov–Smirnov tests. For groupwise comparison, we used Kruskal–Wallis tests. For categorical data, we used Fisher’s test. Post hoc comparisons were made via *z* tests. *P* values below 0.05 were considered statistically significant.

### Sample size

We used all trauma room protocols that were completed in 2016. This period was chosen due to availability of the DA beginning in 2016, and changes in trauma room management in 2017.

### Clinical relevance

Results were assumed to be clinically relevant when a difference of at least 10% was observed for a given comparison.

### Ethical approval and consent to participate

This study involved retrospective analysis of anonymized data collected during regular ED treatment. In relation to the general terms and conditions of the treatment contract of the University Hospital of Magdeburg, this study needed no ethical approval.

## Results

**Fig. 1:**
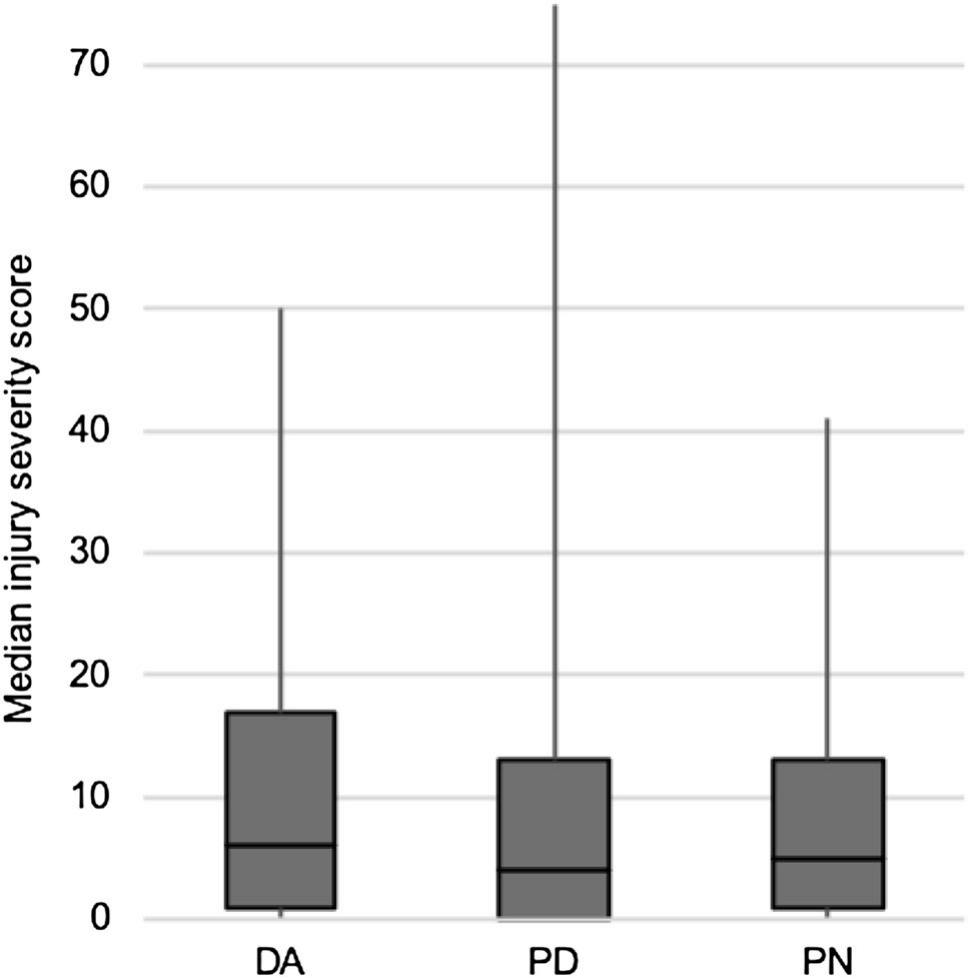
Injury severity score. ISS was calculated from the final diagnosis from in-hospital treatment. The median ISS did not differ significantly between the DA group and PD and PN groups (Kruskal–Wallis test: *p* = 0.721, box: interquartile range, whiskers: minimum and maximum values)

From 01/2016 to 12/2016, the data of 251 trauma room patients (179 male, mean age 42.4 ± 17.9 years; 72 female, mean age 44.8 ± 20.7 years) were analyzed. There were 56 patients in the DA group, 86 in the PD group, and 109 in the PN group. The median injury severity score (ISS) of the entire sample was 4. There were no significant differences in median ISS between the DA group (6; IQR 16) and each of the PD group (4; IQR 13) and PN group (5; IQR 13; *p* = 0.721 by Kruskal–Wallis test; Fig. 1).

### Primary outcome analysis

**Fig. 2:**
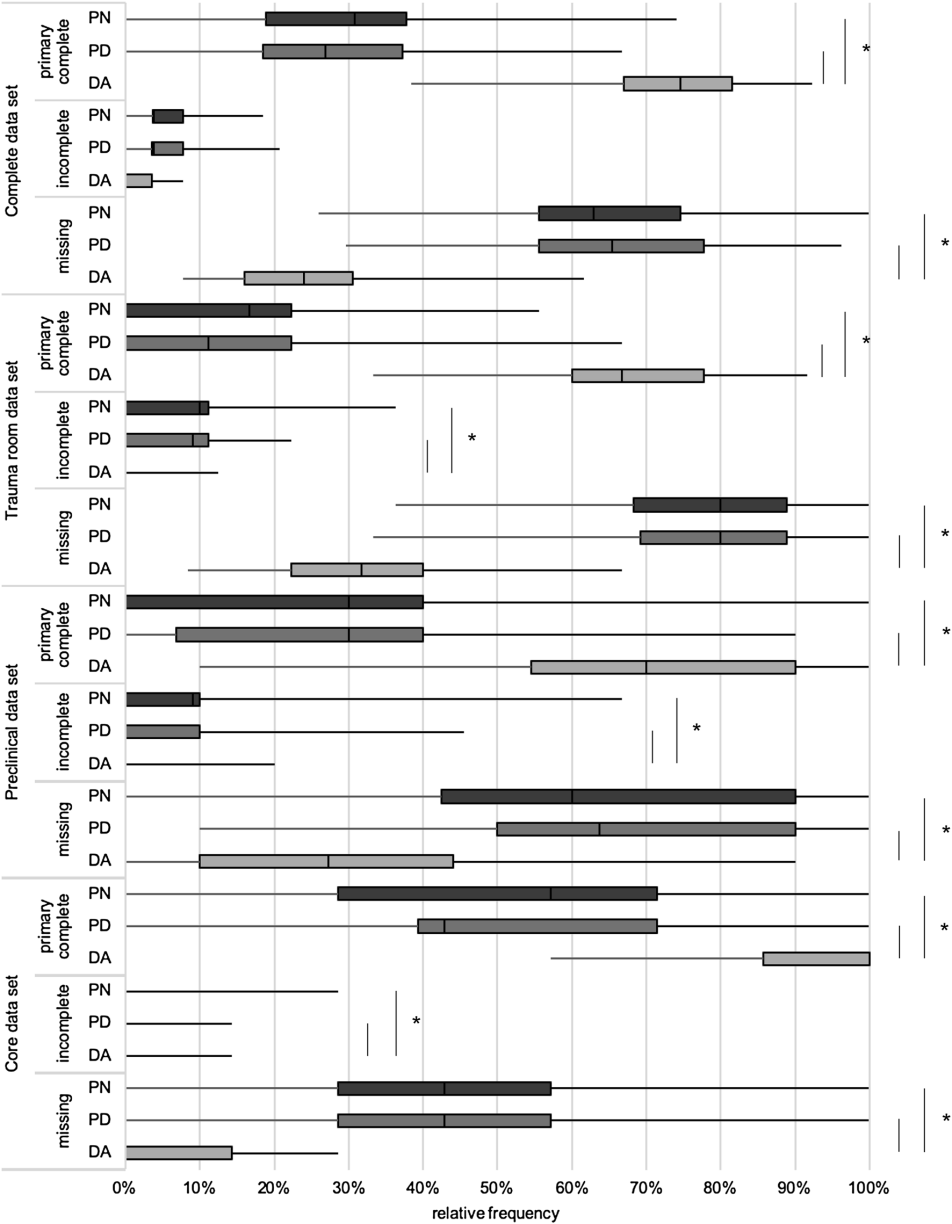
Completeness of acquired data in subcategories and the complete data set. In presence of the DA, frequency of primary complete data was significantly higher for the complete data and the subcategories. The frequencies of incomplete and missing data were statistically significant different among groups. The DA group showed decreased frequency of missing and of incomplete data compared to PD and PN. (Kruskal–Wallis test: *p < 0.05, **p < 0.01, ***p < 0.001; box: interquartile range, whiskers: minimum and maximum values)

**Fig. 3:**
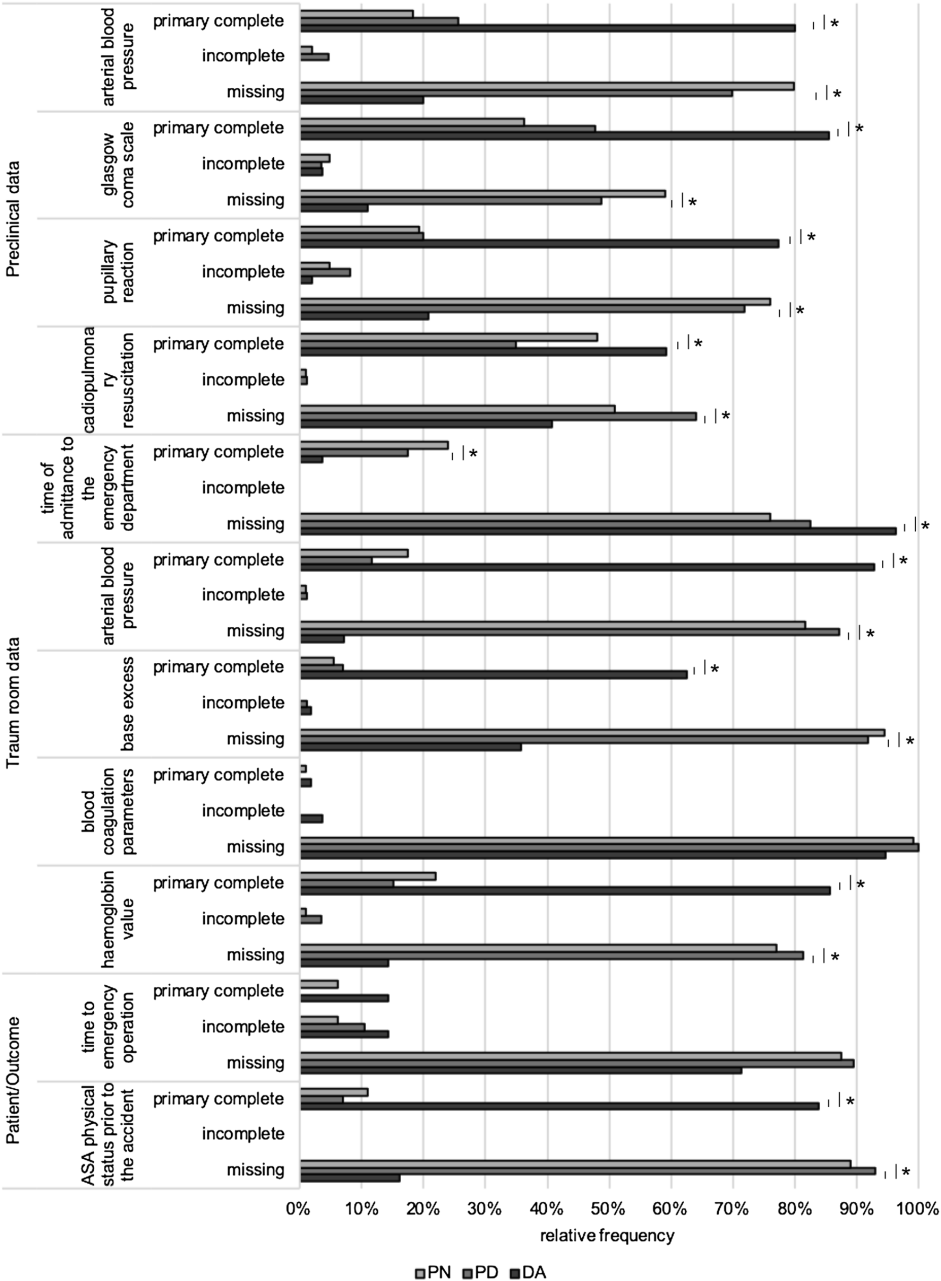
Data completeness of items with high relevance to documentation in the TR-DGU core data set. The documentation completeness of all items except emergency operations and blood coagulation parameters showed a significant increase in the presence of the DA compared to the PD and PN groups (Fisher’s exact test: *p < 0.05)

Regarding all analyzed items, the primary completeness of data differed significantly among DA (74.5%; IQR 14.5%), PD (26.9%; IQR 18.7%; *p* < 0.001), and PN groups (30.8%; IQR 18.9; *p* < 0.001 by Kruskal–Wallis test). Similar results were found for subgroupings by patient core data (*p* < 0.001), prehospital data (*p* < 0.001), and trauma room data (*p* < 0.001; for details see Fig. 2). Incomplete data were collected in the presence of the DA in median in 0.0% with an IQR 3.6%, in 3.8% of cases in the presence of PD (IQR 4.1%; *p* < 0.001), and 3.8% of cases in the presence of PN (IQR 4.0%; *p* < 0.001 by Kruskal–Wallis test). Unrecorded data occurred in the presence of the DA in 24.0% of cases (IQR 14.5%), while the values were 65.4% for PD (IQR 22.2%; *p* < 0.001) and 63.0% for PN (IQR 19.0%; *p* < 0.001 by Kruskal–Wallis test; Fig. 2). The TR-DGU uses a set of items as surrogate parameters for documentation quality and completeness, as shown in Table 1. Here, we observed a significant increase in documentation completeness in the presence of the DA for all items except documentation of blood coagulation parameters and time to emergency surgery. The item cardiopulmonary resuscitation showed a significant increase in documentation completeness for DA compared to PD, but not to PN (for details see Fig. 3).

### Secondary outcome analysis

**Fig. 4:**
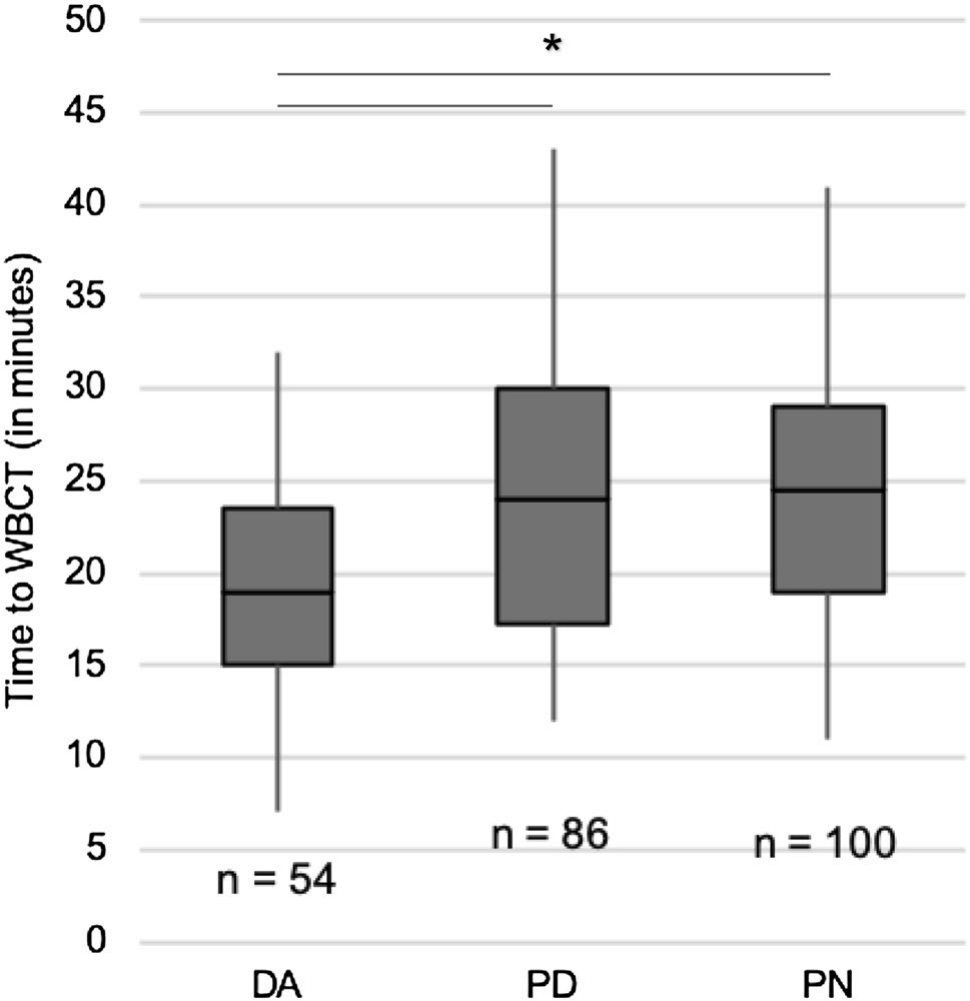
Time to WBCT. Time from admission to WBCT significantly decreased in the presence of the DA compared to the PD and PN groups (Kruskal–Wallis test: *p < 0.001; box: interquartile range, whiskers: minimum and maximum values)

As a secondary outcome parameter, we evaluated the time from admission to the trauma room to whole-body computed tomography (WBCT). The time from admission to WBCT was significantly lower in the presence of the DA (19 min; IQR 8.3) compared to presence of the PD (24 min; IQR 12.8; *p* = 0.007) or PN (24.5 min; IQR 10.0; *p* = 0,001 by Kruskal–Wallis test; Fig. 4).

## Discussion

This study investigated the significance of a qualified medical documentation assistant in the trauma room setting of a German level-one trauma center. We compared the completeness of trauma room documentation conducted by a DA to documentation conducted by a physician involved in the direct treatment of the patient. The primary data quality was significantly better and the time to WBCT was shorter in the DA group. The ISS did not differ significantly among groups.

The TR-DGU is an important tool by which to improve the diagnosis and treatment of severely injured patients [14]. To achieve good data quality, it is important to prospectively register severely injured patients [15] and high-quality documentation quality is crucial [8]. As shown by Grundgeiger et al. [16], a possibility would be digitization of the documentation. They demonstrated that the use of a tablet-based application by a member of the emergency team could improve data quality without compromising clinical performance. However, the data quality reported to date is not optimal, even in level 1 trauma centers [8]. The logical consequence should be the professionalization of primary documentation and data input roles. In the presence of a qualified medical documentation assistant, the primary data completeness was significantly improved in our study. This implies lower cost and effort of entering the data of each trauma room case into the central TR-DGU documentation system. To achieve a better documentation quality, it is further necessary to perform the documentation immediately to the procedures in trauma room. For example, time stamps of the corresponding procedures were not plausible, if recorded retrospectively. As we record the information immediately, one physician is bound documenting the process of trauma room treatment. Therefore, the implementation of a documentation assistant releases one physician from documentation. Thus, this physician is able to support the trauma room treatment. However, this was associated with a shorter time to whole-body computed tomography (WBCT). WBCT plays a crucial role in trauma room diagnostics. It contributes to reducing mortality in patients with severe blunt trauma [17]. Time to WBCT is an important variable. Making available a CT scanner in the trauma room leads to reduced time to WBCT and thus helps reduce mortality among trauma room patients [18].

Regarding the estimated costs of a DA, it is necessary to implement an effectively workflow. In addition to the documentation in the trauma room, the DA in our institution carries out the input in the TraumaRegister DGU® and gathers the informed consent for input in the TraumaRegister DGU^®^ from the patients. Moreover, the DA cares for several clinical studies as study nurse. However, if documentation is carried out by physicians or emergency department nurses, it needs to take into account, that while documenting, they are not involved in the clinical examination and treatment of the patients. Therefore, it is necessary to offset the costs of a nurse/physician to the costs of the DA. Thus, it seemed to be very cost intensive, if this is a physician in comparison to a documentation assistant. The advantage in contrast to a nurse of the emergency department is the input of the information from the trauma room treatment in the TraumaRegister DGU^®^ by the same documentation assistant. In this regard, the DA knows exactly which information was obligatory and needed. Therefore, the effort for retrospective data acquisition is minimized. However, this implementation was established in a level 1 trauma center. Although the results showed improvements in documentation quality, a cost-effective implementation of a study nurse in other trauma centers or hospitals without clinical studies needs another implementation approach.

A limitation of our results is that causality could not be explicitly established; that is, although data completeness was better with a DA, we cannot state definitively that the DA led directly to the improved completeness. Furthermore, even though data completeness was poorer in PD and PN groups, a direct comparison group for the documentation assistant was not available. Moreover, DA was involved only on daytime. Therefore, bias in data interpretation is possible. Due to structural changes in 2017 that affected trauma room management data, data from 2017 and later could not be included. Thus, we only analyze 251 trauma room protocols.

## Conclusion

In presence of a qualified medical documentation assistant, data completeness and time to WBCT improved compared to trauma room treatment with documentation completed by an involved physician. These findings need to be supported by an appropriate prospective randomized study. Nevertheless, implementation of a medical documentation assistant in trauma room treatment appears beneficial for primary data quality concerning information transfer to the attending physician as well as to health services research (e.g., the TR-DGU).

## Data Availability

The datasets generated and/or analyzed during the current study are not publicly available due data privacy rules but are available from the corresponding author on reasonable request.
